# In Vitro Replication of Swine Hepatitis E Virus (HEV): Production of Cell-Adapted Strains

**DOI:** 10.3390/ani13020276

**Published:** 2023-01-13

**Authors:** Giovanni Ianiro, Marina Monini, Maria Grazia Ammendolia, Luca De Sabato, Fabio Ostanello, Gabriele Vaccari, Ilaria Di Bartolo

**Affiliations:** 1 Department of Food Safety, Nutrition and Veterinary Public Health, Istituto Superiore di Sanità, Viale Regina Elena 299, 00161 Rome, Italy; 2 National Center for Innovative Technologies in Public Health, Istituto Superiore di Sanità, Viale Regina Elena 299, 00161 Rome, Italy; 3 Department of Veterinary Medical Sciences, University of Bologna, Via Tolara di Sopra, 50, 40064 Bologna, Italy

**Keywords:** HEV, subtype, HEV-3, A549, isolate, cell culture, ORF2, swine, zoonosis

## Abstract

**Simple Summary:**

The zoonotic virus of HEV-3 genotype is further classified in different subtypes whose biological role is still not clear. Most information available on the virus, both on genetic features and on replicative cycle, had been obtained by studying the genome characteristics and by performing in vitro transfection of permissive cells using replicative clones of the viruses. The lack of an efficient cell culture for HEV cultivation has hampered the study of the virus so far. In the present study, the protocol for HEV cultivation on human A549 lung cells previously established was used successfully to cultivate different subtypes of HEV-3 isolated from pig faeces. The different isolates grew similarly. Sequence analyses was performed on isolates, at different days post infection and in the following passages on cells. Analyses excluded the presence of any insertion in the hypervariable region of the genome, as observed in previous studies, and revealed a few mutations acquired in the viral genomes during the growth on cells. The protocol of HEV-3 cell cultivation was used for a quick production of a high amount of the virus in serum free medium, not requiring further purification. The obtained isolates will be used for future experiments of virus infectivity.

**Abstract:**

The hepatitis E caused by the virus HEV of genotypes HEV-3 and HEV-4 is a zoonotic foodborne disease spread worldwide. HEV is currently classified into eight different genotypes (HEV-1–8). Genotypes HEV-3 and HEV-4 are zoonotic and are further divided into subtypes. Most of the information on HEV replication remains unknown due to the lack of an efficient cell cultivation system. Over the last couple of years, several protocols for HEV cultivation have been developed on different cell lines; even if they were troublesome, long, and scarcely reproducible, they offered the opportunity to study the replicative cycle of the virus. In the present study, we aimed to obtain a protocol ready to use viral stock in serum free medium that can be used with reduced time of growth and without any purification steps. The employed method allowed isolation and cell adaptation of four swine HEV-3 strains, belonging to three different subtypes. Phylogenetic analyses conducted on partial genome sequences of in vitro isolated strains did not reveal any insertion in the hypervariable region (HVR) of the genomes. A limited number of mutations was acquired in the genome during the virus growth in the partial sequences of Methyltransferase (Met) and ORF2 coding genes.

## 1. Introduction

Hepatitis E is an acute viral disease caused by hepatitis E virus (HEV). HEV is mainly transmitted via the oral route [1]. HEV is classified in the subfamily *Orthohepevirinae* of the family *Hepeviridae*, which is divided into different species. Strains affecting humans belong to the *Orthohepevirus A* species, recently re-named *Paslahepevirus balayani* by the International Committee on Taxonomy of Viruses (ICTV URL: https://ictv.global, accessed on 7 November 2022). Strains affecting humans belong to five genotypes of *Orthohepevirus A*: HEV-1–4 and HEV-7.

Genotypes HEV-1 and HEV-2 are restricted to humans; genotypes HEV-3 and HEV-4 are zoonotic, with pigs and wild boar as their main reservoirs, and circulate in developed countries [1]. Genotype HEV-7 has been detected in dromedary and one immunocompromised human case [2]. Other genotypes of *Orthohepevirus A* species have been detected in animals only [3]. In Europe, most of human infections are caused by HEV-3 and HEV-4. The disease is usually self-limiting and asymptomatic. However, it can become chronic in immunocompromised patients, leading to fulminant hepatitis [4].

The HEV-3 and HEV-4 are mainly transmitted by the consumption of raw or undercooked pork and wild boar meat. The infection is widespread in the pig population [5]. In Europe, the HEV-3 is the genotype most frequently detected in both pigs and wild boar [3].

The genome of HEV is a positive-strand RNA coding for three open reading frames (ORF1-3). According to the phylogenetic analysis on a limited number of complete genome sequences, HEV-3 genotype strains are classified into 13 subtypes, together with 6 variants defined to date as unclassified [6]. The knowledge on the significance of the subtypes is still scanty, although a correlation between the different subtypes and the severity of the disease was observed. It has also been hypothesized that the heterogeneity of the sequence could be due to some hosts restrictions, not clearly established so far.

The mechanism of replication of HEV in cells is still poorly known due to the lack of an efficient cell culture system for in vitro replication. The only information available was obtained by animal experimental infections and by using replicative virions produced with cDNA clones of virus genomes transfected on permissive cells [7,8], allowing the establishment of some of the replication cycle stages of the virus. The HEV particle exists in two forms: with envelope (eHEV) or without the lipid membrane (naked, nHEV). The HEV particles released in faeces and present in bile are non-enveloped (nHEV), while eHEV particles with a lipid membrane and ORF3 are present in blood during viremia. Both nHEV and eHEV viral particles can be propagated in vitro in cultured cells [9]. The mechanism of HEV entry into cells is not well known. Some studies employing HEV-like particles have shown that possible mechanisms of virus entry into cells could be via heparan sulfate proteoglycans (HSPGs), internalized via a dynamin-2-, clathrin-, and membrane cholesterol-dependent pathway [10,11,12].

The protocols of HEV cell cultivation developed so far are troublesome, very long and have a scarce reproducibility even when a successful isolation is obtained.

Over the last years, several protocols for virus cultivations have been produced, and some HEV strains have been adapted to grow on cell cultures. However, the strains adapted to grow well on cells in vitro frequently acquired mutations in their genome during their growth. The human HEV3 Kernow C-1/p6 strain was found to be very efficient in infected HepG2/C3A cells after six passages, thanks to acquisition of the sequence of the host S17 ribosomal superfamily into the hypervariable region (HVR) of the HEV genome [13]. Several other studies reported the presence of host genes or part of them in the genome of HEV strains; this presence conferred a greater ability to grow in vitro to mutated strains, compared to the wild type HEV strains [14,15]. Distinct cells lines were used successfully to grow wild types HEV genotypes, including both hepatoma (human hepatocyte PLC/PRF/5, HepG2) and non-hepatoma cell lines (lung cancer cell line, A549), primary hepatocytes, neuron-derived cells, placental cells, and kidney cells [16]. The existence of some extra hepatic manifestations of the diseases, such as neurological disorders, suggests the capability of HEV to infect extra hepatic organs, thus explaining the ability of HEV to replicate in cell lines of non-hepatic origin [17]. The first successful cultivation of HEV was obtained by inoculating human cells with HEV-1 strains derived from positive human faecal samples [18,19]. Later on, cultivation was obtained for HEV-3 and HEV-4 strains from faeces or sera from human patients [20,21,22] and from pigs and wild boar samples [23]. Further attempts were conducted to optimize the protocol of cell culture by applying different growing conditions, varying growth temperatures, Fetal Bovine Serum (FBS) concentration, presence of antibiotics, and presence of dimethyl sulfoxide (DMSO). These different growing conditions, applied during viral growth on several cell lines (PLC/PRF/5, HuH-7-Lunet BLR, A549, and HepG2/C3A), influenced and implemented the efficiency of growth of HEV stains [24,25,26]. Hyper-confluent cell layers were also used in the protocols and are considered an important factor to implement growth of HEV-3 strains [25]. The optimized protocol for cultivation on human A549 cells was developed with HEV-3 human strains [24,25]. 

The cultivation of HEV-3 and HEV-4 strains from pigs and wild boar was obtained using caecum faecal content and other organs on both hepatoma (PLC/PRF/5 cells) and non-hepatoma human cells (A549) [27,28,29].

Cultivation on cells of HEV strains isolated from animals has been achieved less frequently [8]. The hepatocyte-like PICM-19 pig liver stem cell line was observed to support replication of the HEV genotype HEV-3f isolated from faecal samples of pigs experimentally infected with HEV [30] and, very recently, the same protocol has been successfully used for cultivation in PICM-19 cells of the genotype HEV-3f previously isolated from the liver of a naturally infected pig [31].

Protocols for HEV cultivation are promising but, due to the use of distinct cell lines, biological samples for the inoculum, and HEV subtypes, it is difficult to understand to which extent results can be reproducible.

In the present study, we assessed the use of the protocol previously developed for the isolation of human HEV-3 strains on human A549 cells [25] to isolate HEV-3 stains derived from swine positive faecal samples and belonging to subtypes -HEV-3f, HEV-3c, and two distant HEV-3e. In our study, we have also developed a protocol for the generation of viral stocks grown on medium free of FBS, ready to be used in future experiments without additional purification steps.

## 2. Materials and Methods

### 2.1. Faecal Samples and Inocula

Four pig faecal samples (namely: IT-9, IT-12, IT-13, and IT-52) positive for HEV-3, belonging to the subtypes HEV-3e (IT-9 and IT-12), HEV-3c (IT-13), and HEV-3f (IT-52) were used as inoculum for cell cultivation.

For each sample, 10% (*w*/*v*) faecal suspensions in 0.1M Tris-HCl were generated. After vortexing, samples were centrifuged at 10,000 rpm for 30 min and passed through 0.22 µm filters (Sartorius Stedim Biotech, Goettingen, Germany). The resulting supernatants were used for RNA extraction, quantified in genome copies per mL (GC/mL) by Real-time RT-PCR and used as inoculum for viral growth, as described below.

### 2.2. Cell Culture

Lung carcinoma epithelial A549 cells (ATCC^®^ CCL-185^TM^, Manassas, VA, USA) were propagated in Minimum Essential Medium (MEM), supplemented with 10% heat-inactivated fetal bovine serum (FBS; Gibco, Life Technologies, Grand Island, NY, USA), 2 mM l-glutamine, 1% non-essential amino acids (NEAA), 100 U/mL of penicillin, and 100 mg/mL of streptomycin (P/S; Gibco Life Technologies, Grand Island, NY, USA), at 37 °C in a 5% CO_2_ atmosphere. This supplemented MEM, named B-MEM, was used as growth medium. Cell monolayers before infection were detached using 0.05% Trypsin-EDTA, diluted in B-MEM, seeded in a T12.5 flask at 70–80% confluence (2 × 10^6^ cells), and incubated at 37 °C in 5% CO_2_ at least 3 days before viral inoculation.

### 2.3. HEV Isolation

The B-MEM medium was removed and cell monolayers were washed three times with calcium- and magnesium-free phosphate buffer saline PBS- (Ca^2+^ and Mg^2+^ free). The monolayers were inoculated with 500 µL of the faecal suspension (2 replicates for each HEV strains; P0-P2 passages) diluted to ≥1 × 10^5^ GC/mL of the faecal suspension, to reach a 0.1 multiplicity of infection (MOI). Human HEV strain 14-16753 gt 3c (kindly provided by Dr. Mathias Schemmerer, University of Regensburg, Germany) [25] was used as positive infection control. Inoculated cells were incubated for 2 h at room temperature, then the inoculum was removed, and cells were maintained with B-MEM, supplemented with 2.5 µg/mL amphotericin B, 30 mM MgCl_2_ (named MEM-M), and incubated at 34.5 °C in 5% CO_2_ [25].

MEM-M was removed and replaced with fresh medium every 3–4 days for 9 weeks, without replacement of cells, as established in previous protocols [24,25,26].

For serial passage experiments at 63 days (9 weeks) post infection (p.i.), cell supernatants were recovered, subjected to centrifugation at 10,000 rpm for 30 min, passed through 0.22 µm filters (Sartorius Stedim Biotech GmbH, Göttingen, Germany), and then inoculated at MOI 0.1 on fresh A549 monolayers (P1), with the same protocol used in the first step of the isolation. The viral growth was maintained for 9 weeks, and a third passage was performed (P2).

Cells passages were not performed for the whole duration of the experiments. Equally, treated mock cells were included in all passages.

### 2.4. Fetal Bovine Serum Free HEV Stock Preparation and Minimum Infectious Dose

The HEV cell adapted strains obtained at the end of the third passage (P2) were inoculated on fresh A549 monolayers following the previous described protocol. At 3 weeks p.i., the infected cells were detached by treatment with 0.05% Trypsin-EDTA solution, split at 1:2 ratio in T25 flasks, and, after 2 weeks, split again into T75 flasks. After 2 weeks, the medium (MEM-M) was removed, the cell monolayers were washed 3 times with PBS and supplemented with the same growth medium without FBS. For 10 days, the growth medium was collected, stored, and totally replaced with fresh medium (MEM-M without FBS) every day. The cell supernatants collected during the 10 days were pooled together, centrifuged (10,000 rpm for 30 min), aliquoted, and stored at −80 °C.

The HEV-RNA titre was estimated in the produced aliquots as described above. The serum free stock of isolated viruses was used for further passage (P3).

The MID (minimum infectious dose) was calculated by inoculating A549 on T12.5 flasks with 10-fold dilutions (up to 10^4^) of the viral stocks produced, following the protocol for virus cultivation described above up to 28 days, except for the highest dilution, where the growth was followed for 49 days p.i. The experiments were conducted in duplicate with the stocks diluted.

The MID was established at the dilution of viral stocks, at which no HEV-RNA was detected in the cell supernatant at 28 days p.i.

### 2.5. HEV RNA Quantification

Aliquots of 150 μL of the collected cell supernatants were used for RNA extraction. Total RNA was extracted with QiampViral Mini kit (Qiagen, Monza, Italy), following manufacturers’ instructions, with a final elution volume of 50 μL.

Before extraction, a group of 100 samples, randomly chosen during the first passages on cells, were spiked with 10 μL of murine norovirus (MuNoV, 1.5 × 10^5^ TDCI_50_/mL), which was used as a sample process control. MuNoV detection and calculation of recovery rate were performed as previously described [32]. The nucleic acid recovery rate was evaluated before proceeding with HEV analysis and settled with a mean value of 90.2 ± 18.3%.

For HEV quantification, 5 µL of the RNA sample were analysed using the RNA UltraSense One-Step qRT-PCR System (Thermofisher Scientific, Waltham, MA, USA). For quantitative estimation of GC/mL, a standard curve was built as previously described [32]. Cell supernatants (2 replicates) collected at each time point were analyzed in triplicate.

### 2.6. Detection of ORF2 Antigen

The commercially available test HEV Ag ELISA Plus kit (Wantai, Beijing, China) was performed according to the manufacturer’s protocol and used to detect ORF2 antigen in the cell growth supernatant.

### 2.7. Transmission Electron Microscopy (TEM)

Supernatants of infected cells collected during the virus passages were layered onto carbon-coated copper grids to visualize viral particles. After 1 min adsorption, samples were stained with 2% (*v*/*v*) phosphotungstic acid (PTA) adjusted at pH 7.0. Imaging was performed at an acceleration voltage of 100 kV with a FEI 280S transmission electron microscope (FEI Company, Hillsboro, OR, USA).

### 2.8. Immunoperoxidase (IPA) and Immunofluorescence (IFA) Staining

Cells infected at P2 (63 days p.i.) and P3 (viral stocks in growth medium without FBS, 28 days p.i.) were detached from the T12.5 flask by 0.05% Trypsin-EDTA solution, seeded either on 96-well microplates for IPA, or 8-well Nunc Labtech chamber slides (ThermoFisher Scientific Waltham, MA, USA) for IFA, and then incubated at 37 °C for 24 h. Mock-infected cells following the same procedure were used as negative control.

For IPA, cells were fixed with 4% (*v*/*v*) formaldehyde in PBS at room temperature (RT) for 20 min. After washing and permeabilizing with 0.1% Triton X-100 (*v*/*v*) (Sigma-Aldrich, St. Louis, MO, USA), cells were blocked with 2% BSA (Sigma-Aldrich) (*w*/*v*) in PBS (+)/0.05% Tween-20. Cells were then incubated with the mixed primary mouse anti-HEV ORF-2.1 (clone 2.2 Merck, Darmstadt, Germany) and anti-HEV ORF2 monoclonal (Clone 1.6, Merck), or with in-house polyclonal anti-ORF3 antibodies raised in mice, and directed against a recombinant ORF 3 protein (produced in our laboratory) overnight at RT. Following washing with PBS, the goat anti-mouse IgG (H + L) horseradish peroxidase (HRP)-conjugated antibody (Bio-Rad, Hercules, CA, USA) was added for 1 h at RT. Four additional washes in PBS- were performed before adding 50 mM Na-Acetate pH 5.5 and 3-amino-9-ethylcarbazole (AEC, Sigma). Counts of infected cells were carried out by visual inspection (about 1000 cells per well) and recorded as a percentage of positive cells out of the total number of cells.

For indirect IFA staining, the same protocol was applied. Goat anti-mouse IgG (Fc specific)-FITC (fluorescein isothiocyanate) antibody (Sigma-Aldrich, St. Louis, MO, USA) was used to bind primary antibodies. Then, nuclei were stained by SlowFade^®^ Diamond Antifade Mountant with 4 6-Diamidino-2-phenylindole (DAPI, ThermoFisher Scientific MA, USA). Fluorescent images were taken using a Leica DM4000 fluorescence microscope (Leica Microsystem, Wetzlar, Germany) equipped with an FX 340 digital camera and processed with Adobe Photoshop CS4 software (Adobe Systems, San Jose, CA, USA).

### 2.9. NGS Sequencing of Genome Regions of HEV Isolates

Total RNA from 200 µL of cell culture supernatants of infected A549 of passage P0, P1, and P2 at 9 weeks p.i., were obtained using the QiampViral mini kit (Qiagen, Monza, Italy). Total RNAs were subjected to cDNA synthesis using SuperScript III Reverse Transcriptase (Thermofisher, Waltham, MA, USA) following the manufacturer’s instructions suggested for primer specific reverse transcription. The cDNAs were used in PCRs and nested PCRs using the Phusion High-Fidelity DNA Polymerase (Thermofisher Waltham, MA, USA) to amplify 4 genomic regions: three within the ORF1 (Methyltransferase, Met; Hypervariable region, HVR; RNA dependent RNA Polymerase, RdRp) and one within ORF2 (M domain of capsid protein) (Table 1). The cDNA synthesis and the nested PCRs were performed as previously described [28,33,34,35]. The primer pairs used for reverse transcription and amplification are synthesized in Table 1.

The amplicons were extracted from agarose gel 1.5% using QIAquick Gel Extraction Kit (Qiagen, Milan, Italy) with elution in 25 µL of RNase free water. The purified amplicons were quantified using Qubit dsDNA HS (High Sensitivity) Assay kit. The libraries for NGS were prepared using the Ion Plus Fragment Library Kit (Thermofisher, Waltham, MA, USA) following the protocol for the preparation of amplicon libraries without fragmentation. The libraries were sequenced using Ion S5 System on 520 chip (Thermofisher, Waltham, MA, USA).

### 2.10. Phylogenetic Analysis

Sequences obtained by IT-9, IT-12, IT-13, and IT-52 faecal samples and from virus isolates recovered on following passages on cells were submitted to the NCBI-database with the accession numbers: 15 ORF2 OP558154-OP558168; 15 Met OP558120-OP558134; and 11 HVR OP558135-OP558145, 8 RdRp OP558146-OP558153.

Fifteen ORF2 sequences from this study were aligned with 18 HEV-reference sequences and 21 HEV complete genomes from strains isolated on cells (19 HEV-3 and 2 HEV-4). The HEV-4 was used as outgroup (Acc. N.: LC022745.1). The sequences were aligned using Aliview. A Maximum Likelihood phylogenetic tree was built using MEGAX, T93 + G + I model as suggested by model test and 1000 bootstrap replicates.

### 2.11. Sequence Data Analysis

The raw data were trimmed with Trimmomatic tool [36] and the trimmed reads mapped using the Bowtie2 tool to HEV-3 reference sequences [6] as previously described [37] using the Galaxy Aries online analysis interface [38]. The consensus sequences were built using the iVar consensus caller and the variants calling was performed using the iVar variant caller [39], and the SnpEff tool [40] was used for the variants’ annotation.

The consensus sequences obtained for Met, HVR, Pol, and ORF2 regions for each strain cultivated in this study were aligned using Aliview [41] and compared to highlight SNP differences between passages from P0 to P2.

### 2.12. Statistical Analysis

The Shapiro–Wilk test, the Breusch–Pagan test to verify the homoskedasticity, and the Durbin–Watson test for autocorrelation were used to verify normal distribution of viral HEV-RNA titres (Log_10_ genome copies/mL) (α = 0.05). The viral growth rate was calculated as slope of the linear regression model between weeks and the mean of two replicates titres (Log_10_ genome copies/mL). The linear regression was inferred, and the slopes calculated using the emmeans library [42] and compared by Tukey HSD (honestly significant difference) with Bonferroni correction (*p* < 0.05). The statistical analysis was performed using R Ver. 4.1.2. (https://www.r-project.org, accessed on 7 November 2022).

## 3. Results

### 3.1. Isolation of HEV Strains

The isolation of HEV was performed on human lung A549 cell monolayers using as inoculum four swine faecal samples tested positive for HEV by Real-time RT-PCR and collected individually from animals housed in Italian pig farms. Samples IT-9, IT-12, IT-13, and IT-52 were subjected to nucleotide sequencing before isolation and were established to belong to HEV-3e, HEV-3e, HEV-3c, and HEV-3f subtypes, respectively. The two HEV-3e strains displayed 89% of nucleotide identity in ORF2 regions. Such a difference was considered high for two strains belonging to the same subtype; thus, they were both enrolled in the study.

The titres of faecal suspensions were estimated as 6.80 × 10^6^ GC/mL for IT-9, 2.13 × 10^5^ GC/mL for IT-12, 2.04 × 10^5^ GC/mL for IT-13, and 1.67 × 10^6^ GC/mL for IT-52. The suspensions were used to infect cells with the 0.1 MOI (2 × 10^6^ cells) in duplicate. A wide cytopathic effect (CPE) was observed for one of the two inoculum of samples IT-12 at passage P0 and IT-13 at passage P1. HEV detection was not obtained in the supernatant of cells showing CPE, experiments were not further followed. Differently, no CPE was observed in the other experiments, and, starting 1-week p.i., the A549 monolayers changed their morphologies, resulting in a three-dimensional aggregate that persisted over the whole experiment.

**Figure 1:**
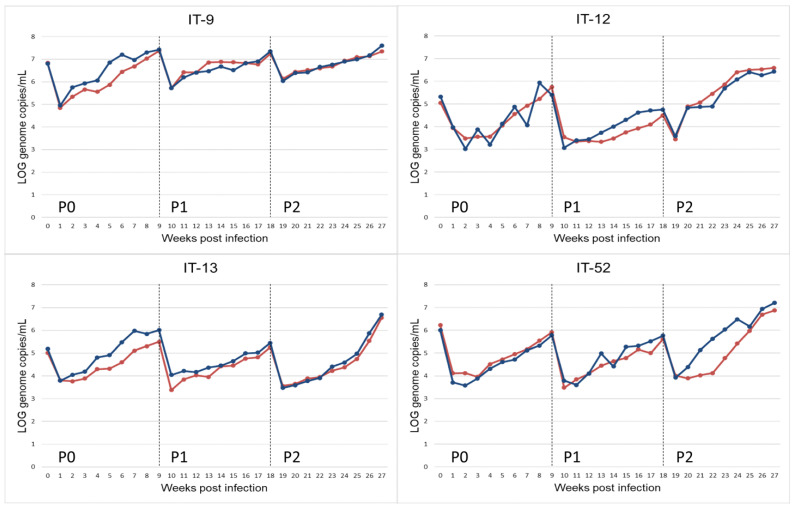
Propagation of HEV-3 strain (HEV-3-e, HEV-3-e, HEV-3f and HEV-3c) on A549 monolayers for replicate r.1 (red) and r.2 (blue). HEV-RNA present in the supernatant of cultured cells obtained during 9 weeks of observation period after inoculation with faecal samples (P0) and the following passages (P1, P2). HEV-RNA titres are measured as Log_10_ copies of genome per mL.

HEV-RNA was found by real time qRT-PCR on weekly collected supernatants from all infected A549 cell cultures confirming propagation of the HEV inoculum. The presence of HEV particles was confirmed by antigen ORF2 ELISA detection on supernatant of day 63 (9 weeks) of passage 0. The titres of HEV were estimated in the collected cell supernatants as 2.42 × 10^7^ GC/mL for IT-9, 2.54 × 10^5^ GC/mL for IT-12, 1.02 × 10^6^ GC/mL for IT-13, and 7.08 × 10^5^ GC/mL for IT-52 (9 weeks p.i.). The suspensions were used to infect new cell monolayers of A549 (P1) for an additional 9 weeks. A third passage on fresh monolayers of A549 (P2) was then performed. HEV-RNA was retrieved in the supernatants, weekly collected during the 9 weeks of cultivation (Figure 1) in all passages (P1–P2). Furthermore, the presence of HEV particles was confirmed by detection of ORF2 antigen by ELISA.

An initial decrease in the viral titre during the first two weeks after the faecal inoculum (P0) and in the following passages (P1 and P2) was observed. This effect could be due to residue HEV-RNA of the input inoculum (Figure 1).

The HEV RNA GC/mL recovered at weeks 9 p.i. for the three passages, corresponding to the last time point of each passage (9 weeks), ranged between 2.7 × 10^6^ and 3.9 × 10^7^ GC/mL (Table 2). At 63 days p.i. of the passages P2, the presence of viral particles was highlighted by TEM visualization. HEV-RNA positive cell supernatants confirmed the presence of viral particles with icosahedral symmetry and dimensions ranging from 25 to 40 nm, as described for the HEV virions.

**Figure 2:**
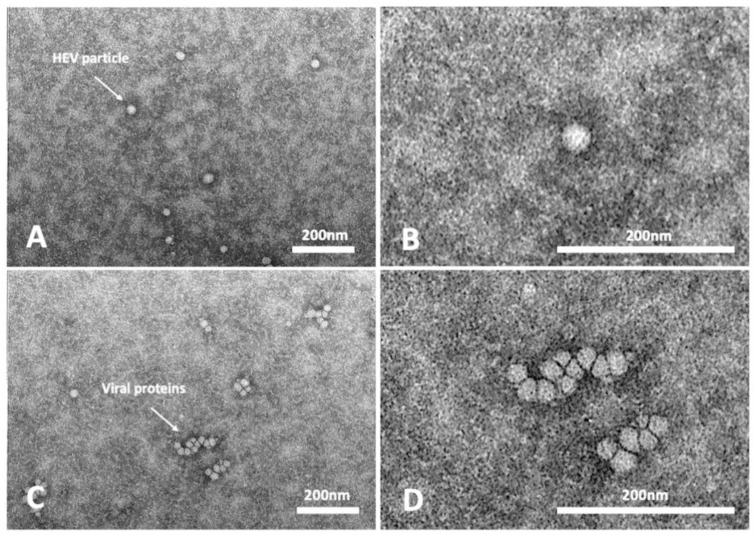
Transmission electron micrographs of infected cell supernatants, positive for HEV-RNA. Representative images of assembled virions (**A**,**B**) and protein aggregates (**C**,**D**).

Many particles, isolates or aggregates, with structural features similar to virions but with no correct icosahedral symmetry and dimensions were also detected (Figure 2). These structures could result from improper assembly of viral proteins.

**Figure 3:**
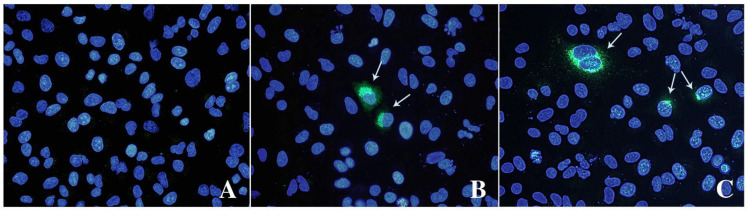
Immunofluorescence analysis of mock (**A**) and infected cells stained with anti-ORF-2 (**B**) and ORF-3 (**C**) primary antibodies. A goat anti-mouse IgG FITC antibody was used to bind primary antibodies. Cell nuclei were stained with DAPI (blue staining). Arrows indicate HEV-infected cells expressing viral proteins. Fluorescent images were taken using a Leica DM4000 fluorescence microscope.

IPA and IFA indicated that virions observed with TEM can replicate and form replicative foci (Figure 3). Both assays revealed a limited number of stained cells, 3–4% of HEV positive cells counted by IPA, with cytoplasmic and perinuclear viral replication and assembly.

**Figure 4:**
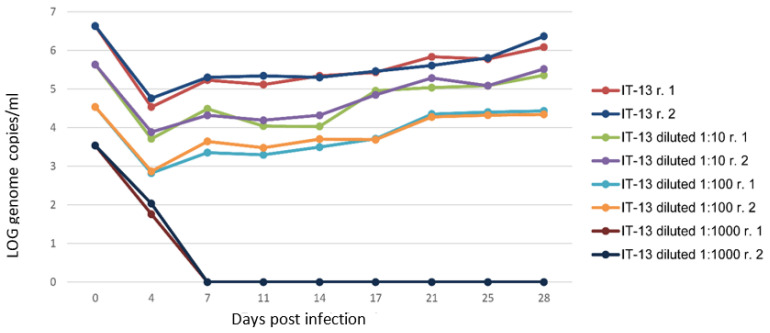
Estimation of minimum infectious dose (MID) of the IT-13 strain. The HEV-RNA in culture supernatants of A549 cells inoculated with serial ten-fold dilutions of HEV stocks was estimated as Log10 genome copies/mL for 28 days p.i. The r.1 and r.2 in legend represent the replicates.

Viral stocks of the different subtypes recovered from cell supernatant at 9 weeks p.i. of the P2 passages were used to infect a new monolayer of A549, and a huge volume of viral stock was obtained during several weeks in the supernatant of cells adapted in growth medium without FBS, as described in Materials and Methods. The titre of the viral stocks was estimated as GC/mL in the supernatant of infected cells (1.50 × 10^6^ GC/mL for IT-9, 2.04 × 10^6^ GC/mL for IT-12, 2.15 × 10^6^ GC/mL for IT-13, and 1.30 × 10^6^ GC/mL for IT-52). These stocks were used to infect new monolayers of A549 (P3), following the same protocol used for P1, P2 passages. The use of additional PBS-washes during medium refresh in the first 2 weeks of cultivation shortened the time of viral inoculum removal (Figure 4), compared to what was observed in previous experiments without these additional washes (Figure 1).

The produced viral stocks grew faster compared to strains obtained from P0, P1 and P2 passages (Table 2). The viral stock reached a 10^5^–10^6^ GC/mL in 3 weeks; differently, the P1 and P0 required several weeks (9 weeks p.i.).

To estimate the minimum infectious dose, A549 cell monolayers were inoculated with 10, 100, and 1000 ten-fold dilutions (10^5^–10^6^ GC/mL undiluted inoculum depending on the isolates) of the produced viral stocks (in MEM-M medium without FBS) following the conventional protocol for virus cultivation. HEV RNA was not detectable in any of the supernatants from cells infected with a starting inoculum lower than 10^3^ GC/mL (Figure 4), following the analyses on cell supernatant up to 49 days p.i. (dilution 1:1000). Differently, viral growth was observed with the other virus dilutions, and revealed that the amount of virus HEV (estimated as GC/mL) released in the supernatant depended on the copies of GC of the starting virus used for the inoculum (Figure 4).

In order to evaluate possible differences in the viral growth among the cultivated subtypes, a statistical analysis was performed comparing slopes of the linear regression models within each passage, between passages of each strain, and in the cultivation on a new monolayer of A549 with the viral stocks produced in growth medium without FBS (P3). The analysis showed that the observed differences on the kinetics of growth of the isolated HEV-3 strains could not be associated either to the subtype of the strain or to the passage of infection.

The comparison between strains within each passage showed no statistically significant differences in P0 and P3, higher viral growth rate for the IT-52 strain with respect to the other strains at P1 (*p* = 0.02), and a significantly higher growth rate for the IT-12 strain with respect to IT-9 at P2 (*p* = 0.01).

In addition, the comparison between passages for each strain showed different results for different strains (Figure 1). In detail, the IT-52 strain at P2 had a significantly higher growth rate than at P0 and P1 (*p* = 0.002 and *p* = 0.003, respectively), the IT-12 strain at P2 showed a higher growth rate than at P1 (*p* = 0.003), and the IT-9 strain at P0 presented a higher growth rate than at P1 and P2 (*p* = 0.0001 and *p* = 0.0002, respectively). Finally, the growth rate for the IT-13 strain at P0 was higher than at P1 (*p* = 0.008), and, for the same strain, the growth rate at P2 was higher than at P1 (*p* = 0.006).

### 3.2. Mutational Characteristics of Isolated Strains in Consecutive Passages

**Figure 5:**
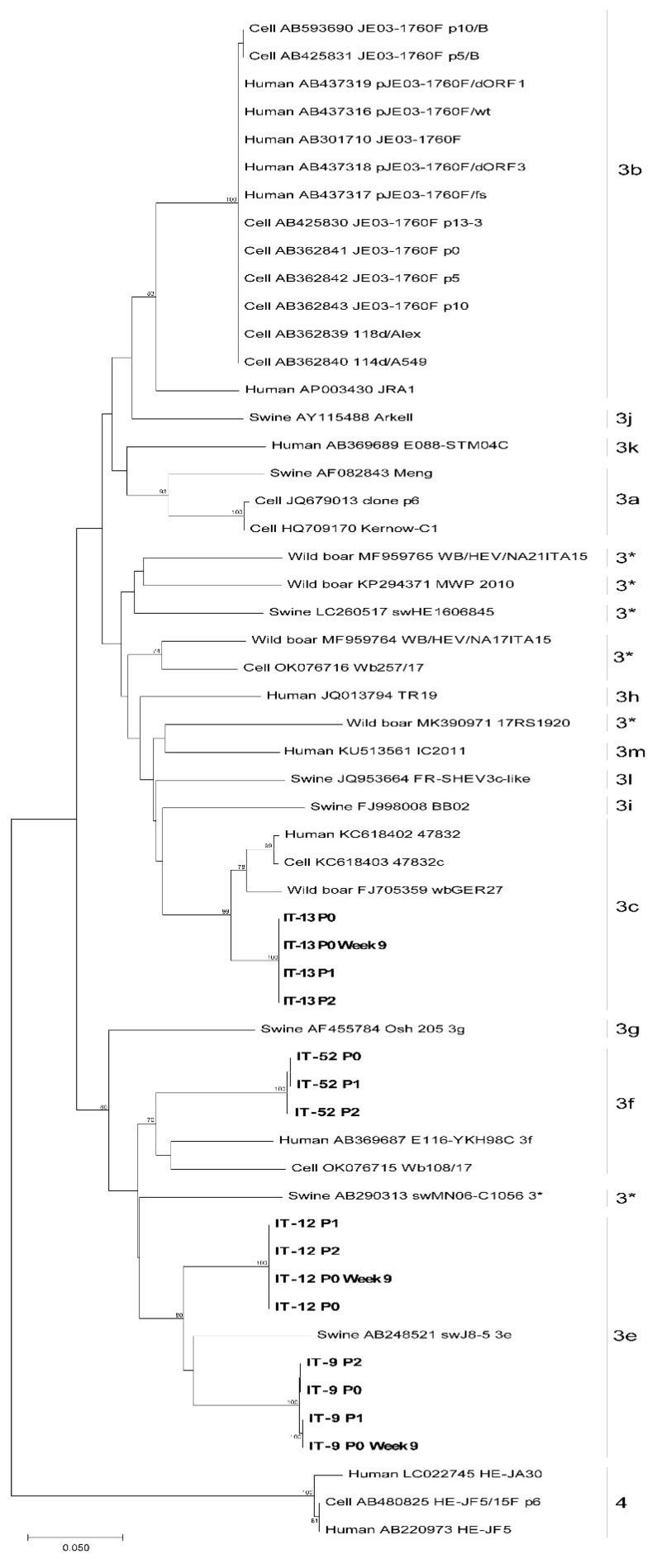
Maximum Likelihood phylogenetic tree built with 15 HEV ORF2 partial sequences obtained from this study (indicated in bold), 18 HEV-3 reference sequences, 21 HEV from strains isolated on cells and one HEV-4 sequence as outgroup. 3*: unassigned subtypes.

Viral RNA obtained from faecal suspensions of the four starting inoculum and from the supernatants of the infected cells from P0 and P1, collected at 63 days p.i. of each passage (week 9), were used to synthetize cDNA subjected to PCR amplifications of the four short genome regions and subjected to sequencing by NGS. For each strain, at least one genomic region was sequenced. The phylogenetic analysis (Figure 5), conducted with the short fragment within the ORF2, confirmed the established subtypes: IT-9 and IT-12 were typed as HEV-3e, IT-13 as HEV-3c, and IT-52 as HEV-3f.

Table 3 shows the mutations detected over three out of four sequenced regions (Met, HVR, ORF2), since RdRp fragment sequences were identical (100% nucleotide identity) in each HEV strain analysed. The differences between the HEV strains in faeces (inoculum) and in its progenies after cell cultivations in the first (P0), second (P1), and third (P2) passages (at 63 p.i. of each passage) were evaluated. Overall, only a few mutations were observed comparing to the original sequence up to the last passage P2: two for the IT-52 and IT-9 strains and one for the IT-12 strain, while no mutations were highlighted for the IT-13 strain.

Within the ORF1, no mutations were observed in Met region of the IT-9 and IT-13 strains, either in P1 or in P2 at 63 days p.i. The IT-52 and IT-12 strains showed one non-synonymous and one synonymous mutation in the Met region, respectively observed at week 9 p.i. of P1 and P2.

Within the HVR region, the IT-9 strain reported a non-synonymous mutation detected at 63 days p.i. of P2, while the IT-52, IT-12, and IT-13 strains showed no mutations.

The ORF2 fragments of the IT-12 and IT-13 strains showed no mutations, while the IT-52 and IT-9 strains revealed one synonymous mutation each, both at week 9 of P2.

The IT-52 wild-type strain (P0) showed a C in positions 189 (Met) and 6181 (ORF2), with a transition to T at the last passage (P2), leading to the exchange of a Leucine to a Phenylalanine (L62F) in the Met region, and no amino acid substitution in ORF2 (A338).

The IT-12 wild type strain had a T in position 316 (Met), and a transition to C (N97) at day 63 after inoculation was observed, fixed in the next passages.

The IT-9 strain showed a G in position 2326 (HVR) and a C at position 6351 (ORF2), at P0, which showed a transition to A (P767) and a transversion to A (A392) at P1, respectively.

Based on the number of days between passages and the mutations observed among isolates over the four regions sequenced (1236 nt), the mutation rate was calculated and estimated between 4.9 × 10^−3^ and 6.2 × 10^−3^ nucleotide substitutions per site per year.

## 4. Discussion

The lack of a valid protocol for cell cultivation of HEV has hampered the study on the virus cycle of replication, response to drug treatments, estimation of infectious dose, and the survival of the virus in the environment and in food subjected to common treatments for safety. This gap of knowledge has limited the study on HEV and the associated risk so far [43].

For the aforementioned reasons, in the last years a great effort has been performed to ameliorate the protocol for HEV cultivation in order to obtain the missed information on the virus.

The virus belonging to HEV-1-4, of both human and animal origin, can be cultivated, but the protocol is laborious: a high titre is required for the inoculum and a time-long growth is required to reach a high titre. Due to the high heterogenicity of HEV-3 strains and the existence of different subtypes, the research results obtained by cell cultivation could be different [8].

In our study, we investigated whether four different strains belonging to HEV-3e, HEV-3f, and HEV-3c subtypes from faeces of naturally infected pigs could replicate on established cell lines of the human lung cell line A549. We succeeded in the cultivation of the different strains and in their propagation following the previous developed protocol [25]. The cell cultivation was still troublesome, but some limits were overcome. The A549 infected cells could be easily propagated by splitting them or by freezing and thawing the infected cells in new flask; the infected cells maintained their ability to release infectious virions in the supernatant, allowing the production of a greater amount of virus. The cells, after several weeksin the same flask, subjected to continuous replace of medium but kept in the same flask, changed their morphology. The infected cells probably became chronically infected and able to continuously produce the virus.

The use of hyper-confluent cells, not replaced during the viral growth for several weeks, was described in several protocols [24,25] with different cell lines [26,44]. As established, the hyper confluent monolayer is a key factor that improves culture yield of HEV at a growth temperature of 34.5 °C [25,26,44].

It was shown that only over confluent cell layers forming three-dimensional structures supported HEV replication, while common monolayers did not, probably because the cell differentiation and the presence of closer contacts between cells causes a greater susceptibility to HEV infection [25]. Moreover, the establishment of cellular autophagy processes allows degradation and recycling of cellular components [45]. Notably, we could obtain infectious viruses by collecting, for several weeks, the supernatant of the infected cells adapted in growth serum-free medium. The advantage was the production of viral stocks in growth medium without FBS that can be directly used for future experiments without any step of purification. Otherwise, in our hands, the amount of assembled virus after purification by caesium chloride is very low, different from the results obtained by other authors [46]. The four HEV-3 strains produced without FBS were able to infect new monolayers of cells (P3), reaching a high titre of virus in the supernatant in a few weeks (3 weeks p.i. were monitored) and the MID of the viral stocks was estimated between 10^3^ and 10^4^ GC/mL, like previously described with human HEV-3 strains [25]. In this study, well-reproducible results were achieved by performing several replicates of the growth on cell culture using the P3 viral stocks stored at −80 °C in growth medium without FBS as starting inoculum (Table 3).

Over the whole study, the presence of the virus in the supernatant of infected cells and its titre was evaluated by RT-qPCR of the ORF2 and expressed as GC/mL. The presence of infectious virus corresponding to the HEV-RNA was estimated by immunostaining the ORF2, coding the viral capsid protein, within the infected cells. The obtained results showed that ORF2 antibodies only stained a limited number of cells (3.5–4%). It can be hypothesized that an inhibition of ORF2 translation could occur in host cells. In a previous study, using reverse genetics with permissive HEV genomes, less than 10% of the cells produced the antigen, and this number remained stable, suggesting that the produced virions, even if infectious, as proved by the successful infection of a monkey with the lysate of infected cells, were unable to infect the remaining cells [47]. A difference of ~1–2 log between the number of infected cells stained by anti-ORF2 and the genome copies of RNA obtained in the supernatant of infected cells was detected, confirming that only a fraction of HEV-RNA correspond to infectious particles or to the production of ORF2 antigen [48].

Furthermore, Montpellier et al. [49] described that the ORF2 capsid protein is massively produced, but only a small fraction is assembled into infectious particles [48]. Our results may also suggest that either only a small fraction of ORF2 is assembled, or only a limited number of cells during the experiments become permissive to HEV infection and replication, overcoming the inhibition of the ORF2 translation, or of post-translational processing, and allowing the correct assembly of the virions [48,49].

The absence of correspondence between viral RNA and infected cells was also reported for other viruses. In a recent study on SARS-CoV-2, it was demonstrated that the characteristic ratio between RNA genome copy measurements and TCID_50_ measurements is about four orders of magnitude but can vary between three and five orders of magnitude [50].

Our experiments highlighted a possible inoculum dose-dependent viral growth, in terms of GC/mL titre reached several weeks after the infection. The lower the starting inoculum, the lower the amount of virus produced. It was hypothesized that a higher viral titre in the starting inoculum provides a higher probability to have the virus with the necessary genome mutations for a more efficient replication [17]. To investigate this aspect, we sequenced four partial genome regions, including those implied in cells binding (ORF2), virus replication (Rdp; Methyltransferase), and the HVR, which varies in sequences and length among strains [51].

We observed very few changes in the isolates between passages (0–2 mutations) in both faecal samples used for the inoculum, and in the virus isolates in the following passages during cell cultivations. Nevertheless, since we did not analyse the sequence of the whole genomes, it is difficult to discuss if other mutations occurred during the growth on the cells.

Previous findings suggested that insertions in the HVR can be present in the genome of cell-adapted HEV strains [14], conferring a greater efficiency of replication in cells or in samples from chronically infected patients [52].

However, in the isolates of this study, no changes in the HVR were observed, confirming that other factors could be involved in cell adaptation [28].

## 5. Conclusions

The protocol of cell cultivation can be applied to different subtypes without any significant difference in the growth efficiency; the protocol developed by Schemmerer et al. [25], was used to produce a high amount of virus in growth medium without FBS as ready to use viral stocks for future experiments.

The study has several limitations: only three HEV-3 subtypes were tested, while tests on additional strains would be needed to generalize our statement; additionally, full HEV genome sequencing of the isolated strains would help to understand if any other region of the genome could be subjected to mutation during cell passages.

## Figures and Tables

**Table 1:** Primers and genome regions amplified by RT-PCR.

Gene	Region	Primer		Amplicon Length (bp)	Reference
ORF1	Met	HEVORF1con-s1 Fw	First-PCR	418	[33]
		HEVORF1con-a1 Rw			
		HEVORF1con-s2 Fw	Nested-PCR	287	
		HEVORF1con-a2 Rw			
	HVR	HVR_2125_F	First-PCR	343	[28]
		HVR_2468_R			
		HVR_2133_F11	Nested-PCR	307	
		HVR_2440_R1			
	RdRp	ISP-4232A	First-PCR	344	[34]
		ISP-4232B			
		ISP-4232C			
		EAP-4576A			
		EAP-4576B			
		EAP-4576C			
		ISP-4232A	Nested-PCR	329	[34]
		ISP-4232B			
		ISP-4232C			
		IAP-4561A			
		IAP-4561B			
		IAP-4561C			
ORF2	M domain	HEV40 Fw	First-PCR	506	[35]
		HEV44 Rw			
		HEV110 Fw	Nested-PCR	458	
		HEV41 Rw			

**Table 2:** Recovered Log_10_ GC/mL of HEV-RNA from first cell cultivation of virus from faeces (P0), following passages P1, P2 and P3 (viral stocks on growth medium without FBS) obtained from cell culture supernatants at different time points. The values of the two replicates (r.1, r.2) of P0, P1 and P2 are reported, whereas for P3 a mean of five replicates is reported (*).

Strain										
			IT9		IT12		IT13		IT52	
			r.1	r.2	r.1	r.2	r.1	r.2	r.1	r.2
Recovered	P0	Starting inoculum	6.81	6.83	5.05	5.32	5.01	5.18	5.99	6.22
		Week 3	5.66	5.93	3.55	3.56	3.88	4.18	3.88	3.95
		Week 7	6.67	6.96	4.93	4.95	5.11	5.97	5.12	5.16
	P1	Starting inoculum	7.36	7.41	5.40	5.74	5.49	6.01	5.77	5.91
		Week 3	6.41	6.43	3.37	3.44	3.03	4.17	4.09	4.10
		Week 7	6.82	6.83	3.92	4.62	4.76	4.81	5.15	5.31
	P2	Starting inoculum	7.34	7.59	6.59	6.42	6.55	6.69	6.87	7.02
		Week 3	6.41	6.51	4.87	5.06	4.22	4.37	4.02	5.12
		Week 7	6.99	7.08	6.40	6.50	4.74	5.15	5.97	6.16
	P3 *	Starting inoculum	6.3		6.56		6.3		6.1	
		Week 3	6.83 ± 0.81		6.31 ± 0.82		5.74 ± 0.16		5.92 ± 0.64	
		Week 7	6.36 ± 0.04		6.40 ± 0.03		6.51 ± 0.02		6.44 ± 1.30	

**Table 3:** Mutations observed on sequences of three genomic regions (Met, HVR and ORF2) of HEV isolates obtained after cell cultivation at 9 weeks p.i.

Strain	Passage	Met		HVR		ORF2		Mutation Rate
		nt *	aa **	nt	aa	nt	aa	
IT9	P1	-	-	2326G > A	P767	6351C > A	A392	4.9 × 10^−3^
IT12	P1	316T > C	N97	-	-	-	-	5.9 × 10^−3^
IT52	P2	189C > T	L62F	-	-	6181C > T	A338	6.2 × 10^−3^

* nt: nucleotide change; ** aa: amino acid change; - no mutations detected.

## Data Availability

Not applicable.
